# A Retrospective Single-Center Study in 20 Patients With Midline Nasal Masses: Which Site Has the Highest Risk of Recurrence?

**DOI:** 10.1177/00034894241300801

**Published:** 2024-11-29

**Authors:** Miray-Su Yılmaz Topçuoğlu, Peter K. Plinkert, Angelika Seitz, Ahmed El Damaty, Heidrun Bächli, Ingo Baumann

**Affiliations:** 1 Department of Otorhinolaryngology, University Hospital Heidelberg, Heidelberg, Germany; 2 Department of Neuroradiology, University Hospital Heidelberg, Heidelberg, Germany; 3 Department of Neurosurgery, University Hospital Heidelberg, Heidelberg, Germany; 4 Department of Neurosurgery, University Hospital Köln, Köln, Germany

**Keywords:** midline nasal masses, nasal dermoid sinus cyst, nasal surgical procedures, nasocranial approach, outcome, recurrence

## Abstract

**Objectives:**

Midline nasal masses are rare and challenging for surgeons. This study examined the site with the highest risk of recurrence following midline nasal mass excisions.

**Methods:**

Surgical outcomes were retrospectively reviewed following excision of midline nasal masses between 2010 and 2022 in the predominantly pediatric patient cohort. The primary outcome measure was the recurrence rate.

**Results:**

Overall, 22 nasal masses were resected from 20 patients. Of these masses, 16 were nasal dermoid sinus cysts (NDSC), 2 were hamartomas, 1 was an epidermoid cyst, and 1 was a mature teratoma. Five of the nasal masses were classified as intracranial lesions, 11 were classified as intraosseous lesions, and 6 were classified as superficial lesions. The open rhinoplasty approach was chosen in 65% of the surgeries. For the intracranially extended lesions, a combined nasocranial approach was performed. Four revision surgeries were performed due to superficial recurrences at the nasal dorsum of lesions, that were primarily classified as intraosseous lesions.

**Conclusions:**

All recurrences had a superficial extension and were easily excised. Intraosseous NDSC have the highest risk of recurrence, but conversely, they also occur most frequently.

## Introduction

Congenital nasal midline lesions in mainly pediatric patients include a broad spectrum of possible differential diagnoses. Nasal dermoid sinus cysts (NDSC) are the most commonly occurring benign congenital nasal midline lesions.^1-8^ They have an incidence rate of 1 in 20 000 to 1 in 40 000 births.^1-3,7-12^ NDSC originate from ectodermal tissue and contain adnexal structures such as hair follicles or sebaceous glands.^1,4-6,13-15^ The cause of NDSC is believed to be a failure in the separation of ectodermal and neuroectodermal tissue during embryological development, resulting in the embedding of epidermal tissue beneath the skin.^9,13,16-18^

Possible complications are recurrent painful inflammations that can lead to abscess formations, meningitis, osteomyelitis, or brain abscesses, particularly in cases where NDSC reach the intracerebral area.^1-3,5,9-13,17,19^ Additionally, nasal masses can damage the growth processes of nasal structures^1,6,8,10^ and may lead to aesthetic impairments.^11^ Surgery should be performed early in life during an infection-free period^3,6,7,9,10,13,16,17,20,21^ to prevent unpredictable and potentially life-threatening infectious complications.^2,5,6,15^ Complete excision of the mass is essential as tissue remnants can lead to recurrence rates ranging from 50% to 100%.^5,7,11-13,15,18,22^

The treatment aims to achieve complete resection of the lesions while minimizing recurrence rates and complications, and achieving good aesthetic results.^3,7,9,10,13,14,18^ Recurrences are the main problem in treating midline nasal masses, particularly NDSC. This study aims to assess which site of midline nasal masses and NDSC, respectively, presents the highest risk of recurrence.

## Materials and Methods

### Recruitment and Inclusion Criteria

A retrospective examination was conducted on patients with midline nasal masses who underwent at least 1 surgery at our department of otorhinolaryngology between 2010 and 2022. A data query was conducted for the disease codes J.34.8 (“other specified disorders of nose and nasal sinuses”) and/or Q18.8 (“other specified congenital malformations of face and neck”) according to the International Classification of Diseases, 10th Revision, and for surgeries encoded with 5-219.0 (“extirpation of a nasal fistula”) according to the national code system for surgical procedures. Eligibility for the study’s inclusion criteria was double-checked for all cases (radiological and histopathological evidence of a midline nasal mass, surgery performed at our department, written informed consent form).

### Ethics

The local ethics committee of the University Hospital Heidelberg approved the study protocol in 2022 under the protocol S-511/2022. The study was conducted in accordance with the current revision of the Declaration of Helsinki of 2013. Written informed consent was obtained from all patients over the age of 18 years, and from the legal guardians of patients under the age of 18 years.

### Surgical Technique

All patients underwent nasal mass excision surgery by 2 senior surgeons of our department of otorhinolaryngology. Surgical success was defined as the patient remaining free of recurrence. The well-known classification of NDSC locations defined by Hartley et al,^8^ namely intracranial-intradural, intracranial-epidural, intraosseous, or superficial, was used to define the extension of the nasal masses seen in our cohort. The surgical approaches used were open rhinoplasty, superficial horizontal incision with oval-shaped lesion resection, vertical midline incision and bicoronar incision, depending on the size, location, and extent of the lesion.^7,8,14,15^ For nasal masses with intracranial extension, combined interdisciplinary nasocranial approaches were performed in collaboration with a senior pediatric neurosurgeon.

### Demographics and Data Collection

The study extracted data from digital medical records on perioperative imaging, medical history, preoperative and intraoperative findings, the surgical approach used, the need for intraoperative nasal reconstruction, the postoperative course, and histopathological results. The recorded data included age at surgery, and gender. The study also assessed the duration of surgery in minutes and the length of hospital stay in nights.

### Statistical Analysis and Reporting

Statistical analysis was conducted using IBM SPSS Statistics for Windows, Version 29.0 (IBM Corp., Armonk, NY, USA, 2022). Prior to statistical measurements, a statistical consultation with the local Institute for Medical Biometry was conducted. Descriptive statistics were presented as absolute and relative numbers. Measures of central tendency, including the median, minimum-maximum range, and interquartile range, were chosen as they are robust against outliers. The STROBE Guidelines were followed as reporting guidelines

## Results

### Demographics

Between 2010 and 2022, 2 senior surgeons from our department of otorhinolaryngology performed 20 surgeries (P1-P20) to excise a total of 22 midline nasal masses. The patients included 8 females and 23 males with a median age of 2 years at primary surgery (Table 1). The primary surgery for nasal mass excision had a median duration of 50 minutes, and patients stayed in the hospital for a median of 2 nights (Table 1).

**Table 1.** Demographics and Surgical Data of Primary and Revision Surgeries.

Feature	20 primary surgeries	4 revision surgeries
Gender	8 females; 12 males	1 female; 3 males
Age at surgery [years]	2 (0-64; 10)	3 (1-20; 15)
Duration of surgery [minutes]	50 (28-360; 50)	26 (9-35; 23)
Length of hospital stay [nights]	2 (0-9; 3)	2 (0-2; 2)

*Note*. The data includes gender, age at surgery, duration of surgery, and length of hospital stay. The values are presented as median (minimum-maximum span; interquartile range). The data on duration of primary surgery was only available for 19 out of the 20 total patients.

Of these 20 patients, 3 male patients and 1 female patient underwent revision surgeries at a median age of 3 years (Table 1). The median duration of revision surgery was 26 minutes, and the median length of hospital stay for revision surgery was 2 nights (Table 1). The follow-up time ranged from 15 months to 14 years.

### Perioperative Imaging

**Figure 1.**
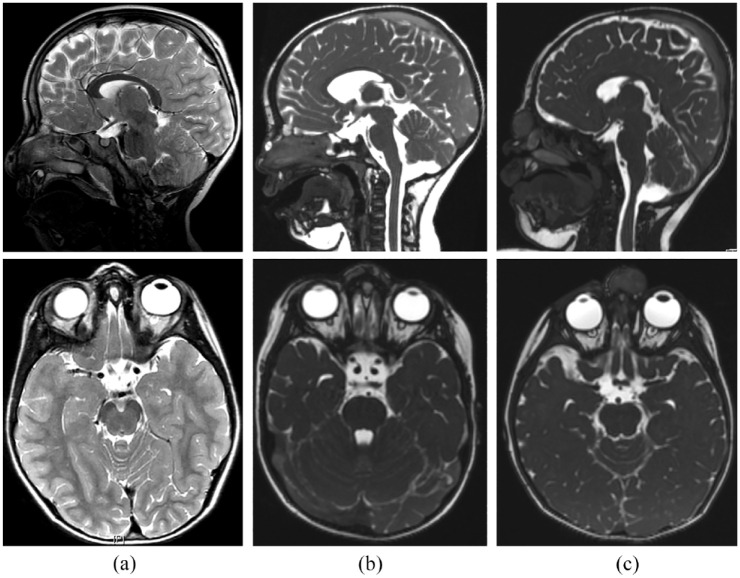
Preoperative imaging. T2-weighted sagittal and axial MRI scans of (a) mature teratoma with intracranial-extradural extension in patient P2, (b) 2 independent nasal dermoid sinus cysts in patient P7 with intraosseous and intracranial-intradural extensions, and (c) glial hamartoma with intraosseous extension in patient P16.

Magnetic resonance imaging (MRI) was performed preoperatively on 16/20 patients (80%) (Table 2, Figure 1). Two patients (10%) underwent both preoperative MRI and computed tomography (CT), while 1 patient (5%) underwent only CT. Only 1 patient did not receive any preoperative imaging as the nasal mass was initially suspected to be an atheroma. However, the final histological result revealed an epidermoid cyst (P18). Follow-up MRIs were performed 6 months postoperatively in 2 cases with outstanding difficult intraoperative conditions.

**Table 2.** Cohort Overview, Preoperative Imaging, and Lesion Characteristics, Including Histological Results, for Both Primary and Revision Surgeries of All 20 Patients (P1-P20).

Patient	Image	Site	Extension	Approach	NS	Reco	Time diff [months]	Site revision	Extension revision	Approach revision	Histology
P1	MRI	Tip swelling	IC-ED	RP	**+**	−	−	−	−	−	NDSC
P2	CT and MRI	Pit and swelling nasal dorsum	IC-ED	RP	**+**	−	−	−	−	−	Mature teratoma
P3	CT	Pit nasal dorsum	IO	RP	−	−	3	Pit nasal dorsum	SU	SI	NDSC
P4	CT and MRI	Pit nasal dorsum	IC-ED	BC	**+**	−	−	−	−	−	NDSC
P5	MRI	Pit nasal dorsum	SU	RP	−	−	−	−	−	−	NDSC
P6	MRI	Pit nasal dorsum	IO	RP	−	**+**	−	−	−	−	NDSC
P7	MRI	Pit nasal dorsum	IO; IC-ID	RP	**+**	−	45	Swelling nasal dorsum	SU	RP	2 NDSC
P8	MRI	Pit nasal dorsum, swelling glabella	SU	SI	−	−	−	−	−	−	NDSC
P9	MRI	Pit and swelling glabella	IC-ED	SI	**+**	−	−	−	−	−	NDSC
P10	MRI	Pit nasal dorsum	IO	RP	−	−	−	−	−	−	NDSC
P11	MRI	Pit nasal dorsum	IO	RP	−	−	−	−	−	−	NDSC
P12	MRI	Swelling medial canthus	SU	SI	−	−	−	−	−	−	Folliculo-sebaceous cystic hamartoma
P13	MRI	Pit and swelling nasal dorsum	IO	RP	−	−	−	−	−	−	NDSC
P14	MRI	Swelling nasal dorsum	SU	RP	−	−	−	−	−	−	NDSC
P15	MRI	Pit nasal dorsum	IO	ML	−	−	−	−	−	−	NDSC
P16	MRI	Swelling glabella	IO	ML	−	−	−	−	−	−	Glial hamartoma
P17	MRI	Swelling nasal tip and nasal dorsum	SU; IO	RP	−	−	−	−	−	−	2 NDSC
P18	-	Swelling nasal dorsum	SU	SI	−	−	−	−	−	−	Epidermoid cyst
P19	MRI	Swelling nasal dorsum	IO	RP	−	−	4	Swelling nasal dorsum	SU	RP	NDSC
P20	MRI	Swelling nasal dorsum	IO	RP	−	−	8	Swelling nasal dorsum	SU	ML	NDSC

*Note*. Extension as classified by Hartley et al^8^ as intracranial-extradural (IC-ED), intracranial-intradural (IC-ID), intraosseous (IO), superficial (SU). The surgical approaches employed were open rhinoplasty (RP), bicoronar incision (BC), horizontal skin incision with oval-shaped resection (SI), and vertical midline incision (ML). Combined nasocranial approach performed with otorhinolaryngology and neurosurgery (NS +). Reconstruction needed (reco +). The time between primary and revision surgery in [months] is also displayed (time diff [months]).

Abbreviations: MRI, magnet resonance imaging; CT, computed tomography; NDSC, nasal dermoid sinus cysts.

### Primary Surgery

Eleven patients presented with a pit at the nasal dorsum (Table 2). One patient had a pit at the glabella (Table 2). The remaining 8 patients presented with a single nasal swelling (Table 2). Two patients (P7, P17) each had 2 nasal masses, which were independent of each other and not connected via a canal. The masses were resected in the same surgical procedure. Overall, 22 midline nasal masses were removed from 20 patients.

Five nasal masses (5/22; 23%) with intracranial extension (4 × extradural; 1 × intradural) were operated on using a combined nasocranial approach in cooperation with the department of neurosurgery (Table 2). Eleven midline nasal masses (11/22; 50%) were intraosseous, and 6 (6/22; 27%) were superficial.

The other surgical approaches used are demonstrated in Table 2.

Out of 20 surgeries, 16 surgeries (80%) were considered successful as there were no recurrences during the follow-up period.

### Complications

In patient P2, who had intracranial-extradural extension of the nasal mass directly at the crista galli (mature teratoma) and underwent a combined nasocranial approach, a cerebrospinal fluid leak occurred postoperatively. The cerebrospinal fluid leak ceased after 3 days of using a lumbar drain without the need for revision surgery. Patient P7 underwent a combined nasocranial approach with a bifrontal craniotomy. Four months postoperatively, the neurosurgeons had to refix the spherical cap due to a loose fit.

### Nasal Dorsum Reconstructions

In 1 case, we performed a septal reconstruction on a 16-year-old patient (P6) due to a defect in the cartilaginous nasal dorsum (Table 2). This defect had the potential to cause a saddle nose postoperatively after wound healing was complete. The reconstruction was carried out using a local cartilage transplant measuring 1.5 x 1.0 cm from the posterior septal parts.

### Revision Surgery

Out of 20 patients with NDSC, 4 patients (20%) underwent revision surgery at a median age of 3 years, ranging from 1 to 20 years (Table 1). Revisions were performed a median of 6 months (3-45 months; IQR: 33 months) after the primary surgery. All 4 cases presented with a superficial recurrence at the nasal dorsum, of which 2 were excised by open rhinoplasty, 1 by a local horizontal skin incision, and 1 by a vertical midline incision. The primary mass location was at the nasal dorsum and the primary mass extension was classified as intraosseous in all 4 cases.^8^

### Histopathology

Table 2 displays the histopathological results. In P2, the histology unexpectedly revealed a mature teratoma (Figure 1). In P12, the histology of a folliculosebaceous cystic hamartoma confirmed the preoperative MRI finding. In P16, the MRI suspected a glial hamartoma or NDSC, the histology finally confirmed a glial hamartoma (Figure 1). After excision of a primarily suspected atheroma, P18 was found to have an epidermoid cyst. Overall, 16/20 (80%) patients had NDSC, with 2 of these 16 patients having 2 independent NDSC. Additionally, 2 patients had a hamartoma (10%), 1 patient had a mature teratoma (5%), and 1 had an epidermoid cyst (5%).

## Discussion

In this study we examined the clinical outcome of nasal mass excisions as an important issue of pediatric skull base- and rhinosurgery. We aimed to find sites and extensions of midline nasal masses that presented to have the highest risk for recurrences.

### Demographics

The median age at primary surgery was 2 years, and at revision surgery 3 years. There was only 1 older patient who underwent surgery at the age of 64 years. He reported having a subcutaneous lesion since childhood. Consistent with previous reports,^2,5,6,8^ the cohort comprised more male than female patients.

The wide span of duration of surgery and length of hospital stay (Table 1) reflects the varying complexities of each single surgery, ranging from simple local excisions to combined nasocranial approaches. Resecting intracranial lesions is naturally more time-consuming and carries greater risks than resecting extracranial masses.

### Perioperative Imaging

It is important to suspect potential intracranial involvement in all cases of nasal masses, as the initial site and presentation of the mass cannot be correlated with such involvement.^5,6^ Therefore, preoperative imaging is necessary to determine the actual extent of the mass and to plan the surgical approach.^1,7,11,13,20^ Some authors have proposed routinely performing both CT and MRI scans.^23^ A CT scan enables a detailed evaluation of the skull base^1,6,9-11,13^ and is suitable for detecting suggestive lesions in patients with intracranial nasal masses, such as a bifid crista galli or a widened foramen caecum.^2,7,14,23^ However, it is important to interpret CT scans cautiously in children under the age of 3 years due to the varying degrees of ossification of the skull base. This can result in misinterpretation of diagnostic findings.^2,7,23^

A high-resolution MRI scan of the skull base is sufficient for preoperative imaging as it can detect intracranial extensions in high quality.^1,4,7,9-13,15,17,19,23^ We recommend using MRI scans alone as the standard, as they avoid additional radiation exposure, which is particularly important for pediatric patients. CT scans should only be used in exceptional cases with difficult anatomical conditions.

Postoperative MRI scans were not routinely performed. However, they were conducted in cases with difficult intraoperative conditions, as suggested by others.^2^

### Lesion Characteristics and Complications

The study data indicate that intracranial involvement (23% of the cases) is not uncommon in patients with nasal masses, although superficial and intraosseous findings were more frequent. This highlights the significance of preoperative imaging in determining the extent of each nasal mass for surgical planning.

The 1 case of immediate postoperative complication with cerebrospinal fluid leak was successfully treated conservatively. This demonstrates that skilled surgeons can perform nasal mass excisions with relatively low risk.

### Nasal Dorsum Reconstructions

Reconstructive surgery is a highly debated issue in pediatric patients, as normal nasal and mid-face development can be compromised.^21^ Various reconstruction techniques have been reported, including fat grafts, temporoparietal fascial grafts, and cartilage grafts from the cavum conchae.^5,21^ In 1 case of our cohort, a local cartilage transplantation was performed using cartilage from the posterior septal parts. This was found to be feasible in the 16-year-old patient as it was not expected to harm the growth zone in these dorsal septal parts.

### Revision Surgery

Previous reports have shown that the site of recurrence was mainly superficial, either at the nasal dorsum or tip.^2,3,6,8,15,22^ However, these reports did not provide detailed examinations on the initial mass extensions.

We found intraosseous NDSC located at the nasal dorsum to be the lesions with the highest risk of recurrence among nasal mass lesions. They all presented as superficial recurrences at the nasal dorsum without osseus involvement. Intraosseous excisions are surgically demanding due to the solid consistency of the nasal bone and the narrowness of the bony canal. To prevent the presence of tissue remnants in the surgery field, it is recommended to perform these surgical steps under visual control by an endoscope and microscope with microsurgical instruments. We have been successful in doing this, as none of the recurrences have been intraosseous.

The fact that the recurrences were superficial may be due to epithelial remnants of NDSC still present at the subcutaneous level. Due to the thin skin of the nasal dorsum, it is not possible to perform a spacious resection in this area without compromising good postoperative cosmesis. Based on our experience with the superficial recurrences of initially intraosseous NDSC, we started to perform a concomitant meticulous, sparing spindle-shaped resection of the nasal skin in this area of interest to remove any potential epithelial remnants of NDSC at the subcutaneous level, simultaneously with the endoscopic and microscopic excisions of intraosseous NDSC in the intraosseous canal up to the foramen caecum. The subsequent consequences of this practice remain to be investigated in future.

The most frequently observed type of recurrence was a superficial recurrence of intraosseous NDSC. It is of relevance to note that in our cohort, intraosseous NDSC were also the most frequently observed lesions. Consequently, it is possible that we may have observed a greater number of recurrences in this group compared to those lesions that occur less frequently. It is crucial to acknowledge the possibility of bias in this context. Other histological entities did not show any recurrence.

Fortunately, superficial recurrences can be easily excised.^8^ Our data supports this, as all revision surgeries remained recurrence-free.

### Histopathology

In most cases, the histopathological results confirmed the radiologically suspected lesion, with NDSC being the predominant histology, accounting for 80%. Unexpectedly, a teratoma was revealed in 1 case through histopathological examination. This finding emphasizes the need for excision of all diagnosed nasal masses. In addition to the risk of infections and abscess formations, findings such as a mature teratoma can potentially become malignant in less than 1% of cases.^24^

### Limitations and Strengths

There were limitations to this study, including its retrospective and single-center design, as well as the small cohort of only 20 patients. These data cannot therefore be generalized, but refer to the results of this study. To improve external validity, multicenter studies need to be carried out in the future. However, given the rarity of nasal masses in the population, studies of the outcome of nasal mass excisions are scarce^5^ and are all retrospective.^8,15,22^ Most studies on nasal masses have relatively low patient numbers.^21^ Denoyelle et al^2^ reported on 36 patients, while Kotowski et al,^5^ Carroll et al,^21^ and Naina et al^7^ each included 25 patients in their studies. Purnell et al^12^ reported on 10 patients. Only 3 reports have included impressive patient numbers, such as Hartley et al,^8^ who studied 103 patients over an 11-year period. Herrington et al^15^ conducted a retrospective study over 44 years with 96 patients. Bradley et al^22^ reported on 74 patients over a period of 22 years.

Our study’s strengths lay in the fact that we report not only on NDSC, but also on other rare nasal mass entities such as mature teratoma and patients with 2 independent nasal masses. To the best of our knowledge, this is the first study that evaluates the lesion sites which are at special risk for recurrences.

## Conclusion

Our findings indicate that intraosseous lesions initially located at the nasal dorsum are the riskiest for recurrences, and they are also the most frequently observed lesion extensions. Intraosseous lesions must be excised with great care to avoid tissue remnants. In cases where there is intracranial extension, an interdisciplinary nasocranial approach is crucial.

The treatment goals are complete removal of the mass, low recurrence rates, and achieving good aesthetic results. Rare differential diagnoses, such as teratoma with a potential for malignant transformation, should also be considered. Due to potential complications, nasal masses are always an indication for surgical excision with preoperative imaging. Prospective and multicenter studies should be conducted in the future to confirm the findings of this study and to assess further predictive values for recurrence.
